# Identity development in multicultural context: Multidimensional self exploration approaches employed by Modern Orthodox women

**DOI:** 10.1016/j.heliyon.2024.e29475

**Published:** 2024-04-09

**Authors:** Lipaz Shamoa-Nir

**Affiliations:** Department of Behavioral Sciences, Zefat Academic College, Israel

**Keywords:** Identity development, Dialogue, Self-exploration, Early adulthood, Religion, Modern Orthodox women

## Abstract

The aim of this study is to examine identity development of Modern Orthodox women as they pursue their studies within a multicultural and multi-faith environment. Content analysis was used to analyze the final papers of undergraduate religious female students in Israel (*N = 47*) who participated in a semester-long dialogue course for Jewish students. The findings revealed three salient themes, suggesting that women's self-exploration developed noticeably within a rich multicultural context: (a) participants engaged in self-reflection by drawing comparisons between themselves and Arab students, leading to an exploration of their religious beliefs and group affiliations; (b) participants embraced their individuality within a multicultural context while balancing their religious duties; and (c) participants critically examined the status of Orthodox women in society, particularly within the family context. These findings highlight the process of identity exploration among Modern Orthodox women, complicated by intersections of religion, gender, and culture. In doing so, this study contributes to the understanding of identity development in multicultural societies.

## 1 Introduction

Collectivist societies prioritize the welfare and needs of the community over those of the individual, often causing individual identities to take a backseat to collective identities. This leads to a situation where individuality is suppressed or subordinated to the values of the group, and deviating from these norms can result in severe social stigma [1]. One such collectivist society is the Israeli Orthodox community, in which core values revolve around religion and family [2]. In this context, questioning either of these values can jeopardize one's sense of belonging within the community. This study investigates identity development among Modern Orthodox women in Israel, aiming to understand how participating in dialogue courses can provide a fertile ground to explore and refine their identities in the context of a socially diverse environment.

### 1.1 Modern orthodox women in Israel

The Modern Orthodox sector in Israel combines traditional Orthodox Jewish observance with an openness to modernity and secular culture. The degree of religiosity and adherence to traditional Jewish law (Halakhah) within Modern Orthodoxy can vary, with members generally upholding the principles of Jewish law but often demonstrating more interpretative flexibility compared to ultra-Orthodox communities [3]. This diversity results in a range of individual beliefs and practices within the Modern Orthodox Jewish community.

For instance, within Modern Orthodoxy, there is a spectrum of approaches to gender roles. While some Modern Orthodox women may adhere to more traditional perspectives, others actively seek gender equality when participating in religious and communal life [4]. This diversity contributes to a dynamic and evolving landscape within the Modern Orthodox community.

In contrast to the Modern Orthodox community in Israel, the transformative impact challenging traditional roles and fostering increased flexibility within the religious and social practices of Haredi Orthodoxy extends to the 'silent' revolution among non-Hasidic Haredi women. In ways that are parallel to Modern Orthodox women, non-Hasidic Haredi women are assuming significant roles, exemplifying a departure from traditional norms within the Haredi community [5]. However, the role of women in Modern Orthodoxy remains a subject of ongoing discussion, with their identity development yet to be thoroughly explored. Given the greater workforce participation of Modern Orthodox women in a broader range of professions, there is a need to explore the coping mechanisms employed by these women to navigate the complex challenges they face when reconciling and synthesizing their roles and responsibilities. This study aims to shed light on how Modern Orthodox women manage and address these intricate issues as part of their ongoing development.

According to the intersection identity theory [6], Modern Orthodox women may occupy a unique intersection of identities as women within a traditional religious society. This intersectionality highlights the complex relationship between their gender and religious roles, as they navigate both traditional gender roles and their pursuit of higher education and career. Moreover, their identity development is further complicated by influences of individualism and feminist thought which they will confront in varying degrees [7,8]. Historically, the prevailing view in the study of Orthodox feminism emphasized a contrast between religion and feminism, assuming that a self-defined feminist would distance herself from religion [9,10]. Those who identified as religious were seen as lacking feminist consciousness, such as by endorsing feminist beliefs, self-identifying as feminist, and participating in collective action on behalf of women or in constant conflict with it [11,12]. However, newer approaches have moved beyond this binary conception, recognizing that Orthodox women can navigate and negotiate these conflicting perspectives [13,14]. Some women reinterpret or choose to emphasize one identity over the other, while others create a synthesis that allows them to integrate their feminist and religious values [5,14]. Moreover, Orthodox feminism in Israel is associated with increased involvement and action in religious life [15,16]. To enhance the comprehension of these matters, this study's objective is to examine the dynamics that Modern Orthodox women negotiate as they maintain a balance between their religious identities on personal, interpersonal, familial, and communal levels.

### 1.2 Religious identity development

Identity develops in a continuous interactive process within and among individuals and their surroundings [17,18]. This process occurs mainly during adolescence, but continues throughout life and is prone to social influences. Furthermore, according to Tajfel and Turner's theory [19,20] the definition of self-identity also relies on one's sense of belonging to a group and is only fully defined in the presence of others [21]. That is, in a self-identification process we sometimes define ourselves in terms of what makes us distinctive from others (personal identity: 'I' vs. 'you'), and sometimes we relate to differences between our group and other groups (social identity: 'we' vs. 'they').

The literature suggests that the social and cultural environments in which individuals are situated can play a significant role in shaping different aspects of their self-identity [22]. Recent research found that already during childhood social understanding is embedded in rich systems of cultural and religious contents, suggesting the role of perceived differences between groups in ethnic identity development in a multi-ethnic society [23,24]. In addition, identity development among adolescents and young people has been tested in various contexts and among various ethnicities and cultures, highlighting the significance of religion and religiosity level in identity development among Christians in Europe and North America [[25], [26], [27]]; Afro-Latinxs [28]; Catholic and Protestant Irish [29]; and Orthodox and secular Jews in Israel [30,31].

Religious identity development is closely related to the broader concept of identity formation, which is a crucial aspect of Erikson's theory, especially during the stage of adolescence (identity vs. role confusion); [17,18]. This stage is marked by the search for a coherent and stable sense of self, including one's values, beliefs, and roles in society. Recent research argued that self-exploration continues during early adulthood (age range 18–25) [32,33], when individuals explore their worldview and ask general questions about the meaning of life, and specific ones about religious values and beliefs. That is, whether their personal worldviews differ from those of their parents [33], and takes account of their belief systems and religious questions, even when individuals do not live in a religious home environment [33]. Given the critical nature of academic inquiry and the diversity of the student and staff populations, it is unsurprising that the university environment is one where young people have been found to make substantial changes to their religious identity [[29], [34], [35]].

Recent research which examined identity development among Modern Orthodox adolescents and young adults in post-high schools educational settings showed both strengthening and weakening of their religious convictions and observances as well as attitudes towards their identity, that is, opposite directions of change in how individuals relate to their cultural or ethnic background [30]. However, Jewish Modern Orthodox women have not been researched much, in particular issues related to personal tension between tradition and modernity that exists in modern-day Jewish Orthodoxy [36]. The present study places its focus on the complexities of Modern Orthodox life for women in Israel and how these women's identities evolve over time, including their perspectives regarding both the Orthodox community and their multicultural experiences.

### 1.3 Exploring identity in dialogues

The literature contends that intergroup dialogue serves as a catalyst, encouraging individuals to actively embark on a process of self-exploration. This dual process encompasses personal reflection, occurring within individuals [31,[37], [38]]. Additionally, it encompasses intergroup dynamics and interactions that unfold between individuals [39,40]. Participation in dialogue courses can have various psychological outcomes for individuals. These outcomes often depend on the specific objectives and content of the course, as well as individual experiences and reactions [41]. Some common psychological outcomes associated with dialogue courses include: reduced prejudice and stereotyping, enhanced communication skills, and improved conflict resolution skills [39,40,42].

Studies in institutions of higher education indicate that participating in a course encourages participants to explore their own and others’ experiences in society [29]. When students engage in dialogue about various social issues, they often question their own preconceptions, challenge their existing identities, and potentially develop a more comprehensive understanding of themselves and others [41]. These findings highlight that participating in dialogue courses, particularly in higher education settings, can facilitate individuals in understanding and exploring not only their own but also others' experiences within society. However, it is important to acknowledge that there is a significant gap in research concerning identify development among specific populations, such as Modern Orthodox Jewish women. This study aspires to bridge this gap by placing its focus on this specific social group. By doing so, it seeks to offer a more comprehensive understanding of the intricate dynamics at the intersection of religious and gender identities. The experiences of Modern Orthodox Jewish women are expected to provide unique perspective in this regard, shedding light on the complexities of identity development.

## 2 The current study

This study focuses on identity development within dialogue courses. A dialogue course is a structured program aimed at challenging student's perceptions of themselves and others to bring about critical self-reflection and positive understandings of identity in a pluralistic context. During the course students actively engage over an extended period in coordinated dialogues around topics such as personal and group identity; conflict resolution; otherness; religiosity and secularity in society; personal and cultural history in light of others, etcetera. At the end of the course students are encouraged to reflect on the dialogues they engaged in, the views of others, and then to consider their own views in light of these experiences.

The college population in which this study was conducted, consists of students from a variety of Israeli religions and nationalities. Among the Jews, there are Secular, Reform, National Religious, Modern Orthodox, Ultra-Orthodox, veteran residents, and new immigrants. Among the Arabs, there are Muslims, Druze and Christians [38]. It should be noted that Israeli society consists of a Jewish majority (73.9 % of the population), and an Arab minority - divided into a number of religious affiliations (18 % Muslim, 1.5 % Christians, and 2 % Druze). The Arab minority is often perceived of as having a lower socio-economic status in terms of education, employment, and finances compared to the Jewish majority. However, the college's objective is to foster intergroup comprehension and mitigate cultural and religious tensions, primarily concerning the conflicts between Jewish and Arab communities in Israel, as well as the Israeli-Palestinian conflict, both of which are discernible in the students' perspectives [38].

Research has shown that identity development can be significantly influenced by multicultural college experiences [43]. Furthermore, a recent study has emphasized the significance of perceived cultural and religious distinctions between ethnoreligious groups in shaping the identity development of students [31]. Expanding upon prior research, this study aims to explore the potential impact of daily encounters in a multicultural environment on the process of identity development among Modern Orthodox women. The objective is to investigate whether and how these women, originating from Orthodox backgrounds, navigate the challenges posed by multiculturalism, and how these encounters contribute to their self-exploration.

To address these questions, the study poses the following three questions.

1. Whether and how the tension between Orthodoxy and modern lives is expressed in the women's papers; especially, what are the issues the women face and struggle with in the course and during their everyday life?
2. How do young Jewish Modern Orthodox female students, attending a multicultural college with ethnoreligious groups on campus, engage in the process of exploring their identities?
3. Do Modern Orthodox women face personal challenges or issues in their interactions with ethnoreligious group members on campus, particularly Arab students, and how do they address these challenges?

## 3 Methodology

### 3.1 Research context

A multi-cultural Israeli college was chosen in which Jews and Arabs mixed daily throughout the campus community. This includes in class and during co-curricular activities. Students of the college were offered the opportunity to engage in an extended, structured course of dialogues aimed at exploring questions of identity over the course of a semester. The course was offered either for Jewish and Arab students, single-gender or mixed gender groups of students, such as Jewish or Arab separate groups or female students, Arab women, or Jewish women. In the mixed groups there were two facilitators, one Jewish and one Arab, and the homogeneous groups had a Jewish or Arab facilitator, depending on the ethnic and religious background of the participants. At the end of the semester students were asked to submit a written piece that focused on their experiences during the dialogue. The students formed the participants of the research and the final course material was used as the qualitative data for this study.

The participants of the current study participated in dialogue for Modern Orthodox Jewish women studying in a special program. The course lasted one semester (six to seven encounters of four academic hours spaced every other week), each dialogue group comprising from 17 to 23 participants. At registration time the academic advisors offered all students the possibility of joining the course for the first academic year. Those willing to participate underwent a short admissions interview. Acceptance criteria included a short simulation to test the student's personal ability to participate in a discourse and some questions to assess motivation to take part in the dialogue course (acceptance rate was 97 %).

The course had a female facilitator, whose specialty was facilitating dialogue on Jewish identity. It comprised workshops in various subjects, concerning identity issues and relationships between religious, secular, and traditional Jews living in Israel. The course included discussion on those topics, and relationships between religion and state, Judaism and democracy, and other personal and social issues (involving tension between religion and modern life), such as morality, relationships between men and women, sexuality, feminism, and women's status in society.

### 3.2 Participants and procedure

The dataset is part of a larger study on students' experiences and attitudes during dialogue courses run by the college to promote inter-cultural understanding and positive understandings of identity in the context of pluralism (Ethics: ZAC #14–2015). This study focuses on sub-set of the data obtained from Modern Orthodox women who participated in the dialogue course. The sample is from four different dialogue courses held in four different semesters during 2013–2015.

Forty-seven undergraduate female students volunteered to participate in the research; all were born in Israel, and were ages 18–25 (Although women of different ages participated in the course, only those in young adulthood were selected for the study) [32,33]. Participants’ religious affiliation was based upon self-definition. Thirty-one women were married, and fifteen were single. All participants volunteered to attend the dialogue course in exchange for academic credit. The participants were recruited through an email that was sent from the department secretary in which, they were informed about the study, the use of their data and all students gave permission for their work to be used for this research. All students were notified of the intention to use their work for research, and written informed consent was obtained from all participants involved in the study. Additionally, the participants were assured that refusal to participate in the research would not affect their grades in any way.

A content analysis was undertaken on summary papers submitted by participants after the course was completed. The final papers were written individually, as part of requirements for course credit. The papers, submitted two weeks after the course ended, included a description of student experiences during the dialogue. Their identities remained confidential, with all demographic information deleted to maintain their anonymity.

### 3.3 Coding procedure and analysis

The final papers included open answers to a number of questions on participants' experiences in the dialogue course. These questions aim to gather participants' reflections and insights on their experiences, and challenges encountered during the dialogue course: (1) why did you join the dialogue course? (2) How did participating in the dialogue course influence you? (3) Can you describe any specific challenges or conflicts you faced in the course? If so, please provide examples. (4) In what ways did the multicultural context of the college influence you? (5) Can you share any insights of positive outcomes related to your experience during the course? In the first stage, the author and another facilitator analyzed an initial set of eight final papers to identify distinct themes and decide which ones to pursue. Initial analysis revealed three themes: addressing characteristics of women who raised religious identity questions, addressing identity questions that were influenced by the dialogue's multicultural environment, and focusing on how to deal with those identity questions. Following qualitative analysis procedures delineated by Bryman [44], and after comparing notes, we developed a coding scheme for the three major themes and the sub-themes. Then the coding process tracked relevant themes in each paper. At the last stage, two research assistants performed a separate blind coding, and we compared the agreement of their codes with the author's codes. The coding results had high reliability; agreement with the author codes was highly reliable (see Table 1 for agreement by sub-theme). The analysis reported below is based on the agreed codes.

**Table 1:** Coding Scheme for Self-Exploration Strategies of Young Modern Orthodox Women in a multicultural context.

Coding Scheme			
Theme	Theme Content	Subthemes	Interater agreement
Arab Student as catalyst for identity development	Participants compare themselves with Arab students while exploring their religious beliefs and group affiliation	Religious diversity openness	96 %
		Group identity boosts self-affirmation	92 %
Embracing uniqueness and navigating religious responsibilities	Participants discussed challenges related to personal, religious, and social obligations leading to self-definition strategies	“Different” is “Unique"	90 %
		“Gatekeeper” of tradition or “Agent of change"?	89 %
Applying critical thinking to analyze women's status in society	Participants referred to their own beliefs and those of others in relation to the status of Orthodox women, especially within the family context	Balancing career and family	89 %
		Gender roles and religious norms	80 %

## 4 Results

The findings demonstrate that participating in a dialogue course can enhance the appreciation for diversity and serve as a platform for self-identity development among young Modern Orthodox women. The content analysis revealed three primary themes that encapsulate the self-exploration strategies of these women within a multicultural setting, as presented in Table 1.

### 4.1 Perceived differences with Arab students as a catalyst to refine social identity

The first theme revolves around participants' identity exploration, which is influenced by their comparisons and contrasts with 'others.' In particular, when comparing themselves to Arab students in the college, participants exhibited enhanced openness to diversity while still adhering to their religious beliefs. This process also led them to re-examine their sense of belonging and contributions to their own group, ultimately strengthening their self-affirmation through group identity expression.

#### 4.1.1 Enhanced openness to diversity while remaining within the framework of religious beliefs

Participants mentioned that they became more open to diverse perspectives and more accepting of students from different backgrounds, for example:

Although I am an opinionated and independent woman, I realized that I have an opportunity here to 'earn' something more than just an academic degree. The meeting with all the Arab students in the library and cafeteria made me think about the differences between us [Jews] and them, and that maybe there is also a lot in common between us […] Maybe it's a pity we did not have a dialogue with them in order get to know their religion better (Dina).

Here Dina points to the cultural diversity in the college, as catalyst for joining the dialogue course. In fact, more than half of the participants mentioned contact with students from diverse ethnoreligious groups as a motivating factor to engage in identity exploration. Some of them even indicated that the college social environment encouraged them to "deal intensively" or "deeply" with their religious beliefs and self-perceptions. Dina also identifies as an "opinionated and independent woman," suggesting that she entered the dialogue course with a mindset that the process may challenge her perceptions and contribute to her identity development.

It should be noted though, that most of the participants (75 %) indicated that their main reason for participating in the dialogue course was the academic credit. However, they mentioned it as "an important framework that is good to have in the college," and were full of appreciation for the "opportunity to choose such a course that can contribute to me personally." Hence, it is not surprising that these women, who chose to study in a multicultural and non-religious college and voluntarily joined the dialogue course, set flexible boundaries and distinctions in the course of everyday life. Indeed, in all the final papers the women addressed concerns and issues they struggled with both in the dialogue course and during their everyday lives, especially issues concerning "religious authority," faith, freedom, and identity.

At the same time, to other women it was important to set clear boundaries regarding the desire to change their worldviews during the dialogue course. For example:

I joined the course with solid perceptions about my life, and I had no need to ask further questions about my identity as a Jewish woman or get ideas from other women. When I have questions about Jewish laws (*Halacha*) I prefer to discuss them with my husband or with the rabbi of our community (Rachel).

As a [Jewish] religious woman, I hardly deal with religious dilemmas in daily life; because life in our community is very comfortable and protected […] it was only when I was a teenager that I had personal questions related to faith (Miri).

It appears that although the women expressed openness in sharing their perspectives, the in-depth exploration of these views, characterized by introspection, was a preference reserved for their religious communities, among individuals whom they admired and trusted. Beyond an individual inclination, these statements may also mirror a broader societal phenomenon found among young people in traditional settings—a reluctance to entertain the possibility of evolving worldviews. Hence, the development of identity for Modern Orthodox individuals involves a negotiation that extends beyond religious-secular values, encompassing the specific social contexts in which these negotiations occur.

#### 4.1.2 Strengthening self-affirmation through group identity expression

This sub-theme underscores the efforts made by participants within the multicultural college setting to assert their sense of belonging to their social group, especially when they faced circumstances that they perceived as potentially threatening or challenging. Approximately 40 % of the participants expressed concerns related to comparing themselves, particularly with non-Jewish students, including the Arab students. These comparisons were not primarily focused on identifying similarities but were more intent on highlighting differences between Jewish and non-Jewish students:

The matter of relating to none-Jews ["*ger*" or "stranger"] is central to Orthodoxy […] None of us [participating in the dialogue course] are deluding ourselves that studying at this college will make us see ourselves and the Arabs as similar […] It is true that there is similarity in a small part of the characteristics, but in general the Jewish religion is different (Nechama).

Nechama's use of the term "ger" ("stranger"), a concept rooted in religious scriptures, appears to be a deliberate emphasis on the differing statuses held by the two groups, Jews and Arabs. This choice of wording suggests Nechama's intention to maintain a distinct self-concept by highlighting a separation from the other group. Furthermore, Nechema's statement reflects a sense of discomfort from trying to reconcile her religious beliefs and her experiences in a multicultural setting with individuals from diverse backgrounds. This explanation is reinforced by another finding, according to which 45 % of participants referred primarily to their social-collective identity as "a worldview which is common to all [Jewish] religious women studying in college." Such social positioning implies that group identity plays important role in how participants perceived themselves and interacted with others in college, as Michal wrote:

The decision to study in a secular college with Arab students was not easy, and it took me a long time to accept it as a positive decision […] After the first year, I thought about quitting my studies. The intercultural encounter can make it challenging to maintain a clear identity. Nevertheless, during the dialogue in a group of Jewish women that share my values, I realized that this experience has contributed to the strengthening of who I am and my self-perception as a Jewish religious woman (Michal).

The above sub-themes suggest that there are two seemingly opposing aspects of self-motivation. Rather than conflicting with each other, they support and facilitate the process of women's identity development in some way. First, in light of the multicultural context in which the dialogue course occurs, women become more receptive to different cultures and viewpoints while still adhering to their religious beliefs and values. At the same time, women become more knowledgeable about their social affiliation as Jewish group members, and how they are positioned within the college cultural context. This increased awareness has led to a greater sense of self-awareness, reinforcing their Jewish-Orthodox collective identity.

### 4.2 Identity dynamics: embracing uniqueness and navigating religious responsibilities

In a complex intersection of religion, culture, and gender, participants in the dialogue course experienced identity development that contributed to their self-definition as Modern Orthodox women. Engaging with diverse students highlighted the significance of understanding their identities and led to the emergence of two self-definition strategies.

#### 4.2.1 Being different is "unique"

As the term suggests, the first sub-theme shows that engaging effectively in dialogue and expressing their thoughts and feelings have contributed to a noticeable boost in the participants' self-confidence. The women referred to their "uniqueness" at home and at work, citing this as the reason why they opted to study in a college with both secular Jews and Arabs, rather than in an Orthodox institution. In this context, they viewed themselves as distinct and special individuals. As a result, around 20 % of the participants emphasized their personal strengths and qualities by using traits and characteristics such as, "determined", "unique" and "exceptional". Few women insisted on presenting themselves as "pioneers" in the process of dealing with a multicultural reality in higher education, and generally as having a high level of self-confidence. Given the choice to study academic studies in a multicultural college (in which there is a relatively large number of non-Jewish students), the women mentioned a feeling of "being part of something that not every religious woman experiences." Furthermore, they also perceived themselves as unique in being "capable of dealing with two worlds," Modern Orthodox world and secular Jewish world. For example, Miri refers to her challenge of balancing the desire for uniqueness with maintaining the status quo as the "juggle of my life." Describing it as a skillful act, Miri sees herself as successful in this delicate balance. These examples of self-definition highlight that these young women were in a stage of self-identity development where they were primarily exploring their individual traits and qualities.

#### 4.2.2 Gatekeeper of tradition or "Agent of change"?

In line with the first sub-theme, this theme concerns the way the participants deal with their significant social role "as educators of future generations." Especially, this strategy involves justifying the need to stretch beyond Orthodoxy's boundaries, yet without creating conflicts or clashing with the religious establishment. Some women (25 %) emphasized that their lives were conducted according to strict religious standards, and some of them defined themselves as "strong religious women." However, while being different, they expressed a desire to bring "a spirit of change" to their communities, while indicating that they were careful not "to stretch the boundaries too much." This pattern found among most married participants and mothers of children was explained by the motivation: "out of responsibility for future generations."

There are many complex issues related to religion in the college […] To choose to study at a secular college was a bold step I took, that led to significant learning, but I'm not sure if it's the right choice for my children. I prefer that they choose a place where they can have friends and people similar to them, so they won't have to deal with questions of kosher dietary laws and modest dress, and instead, focus on their studies. My husband supported my decision, but when it comes to the children, it's a different story […] (Hilla).

Hilla's statement reveals a central theme of self-reflection and decision-making, illustrating the nuanced identity development of Modern Orthodox women who are also mothers. This process involves a dialectical approach, where women express a desire to apply their unique perspectives while carefully preserving the stability of identity elements related to religiosity, relationships, and family. A notable aspect arises concerning choices made for their children. Their hesitancy to expose them to certain options indicates an awareness of raising them in a community with a more insular ideology and education, minimizing exposure to external influences. Despite concerns for their children, these women confidently engage with secular knowledge and influences, drawing strength from their faith and education to navigate challenges in the secular world.

It should be noted that the women's written papers included both self- and social approval, such as: "I feel [a sense of] belonging even though I'm different," and "They [neighbors] accept that I'm different." By addressing the issue of "retaining community allegiance," the women perceived themselves concurrently as having strong affiliation with their communities.

### 4.3 Applying critical thinking to analyze Women's status in society

The college context prompted participants to engage in critical thinking about their own beliefs and the beliefs of others. This process led some women to develop a more nuanced understanding of complex issues related to the status of Orthodox women, particularly within the family context.

#### 4.3.1 Balancing career and family

What about career aspirations? […] I do strive for having a successful career, but it was only after the birth of my firstborn, I realized that in the eyes of the society family must come first. This is how it is with us in religious Zionism […] All religious women have the willingness to compromise career for family (Noa).

Noa describes the compromise she made regarding the tension between home and career, in light of the social demand to be "more at home". This is not surprising, given Orthodox society's pressure to conform to traditional roles, according to which women are expected to give their family priority. Indeed, the participants who mentioned career aspirations (15 %) addressed the work-family conflict only from one direction of the conflict: work interference with family. This attitude is prevalent among women who are not characterized by feminist self-identification and high participation in feminist activism, and may reflect an emergence of social status among these young women. That is, being in the stage of studies and at the beginning of a career, participants' professional identity is in the early stages of development.

At the same time, about a quarter of the participants presented themselves as having an "important job", being "leaders in their communities," and generally as "hard workers." Moreover, few of them addressed their mothers or sisters as a "role model" and as "having a successful career".

#### 4.3.2 Gender roles and religious norms

Even though only four women used the words "feminism" or "feminist," about half (40 %) of the participants referred to at least one discussion or question which relates to gender issues in society, and family life and gender equity. However, instead of trying to bring about change by directly opposing male authority or challenging the existing cultural norms, these women chose to become a part of the existing cultural structures, waiting for the right timing to endorse changes:

The principal of the school does not consult with me as he does with the male teachers […] I know it has nothing to do with me being a substitute teacher but with being a woman, but I am keeping quiet for now until I finish my degree and internship and when I become a permanent teacher […] I will find a way to influence what is done at the school. I have an example of my mother who is now a vice principal at another school (Yael).

It appears that Yael references her mother not solely as a mentor from whom to glean experience, but more significantly as evidence of the triumph of women's efforts for workplace equality, and perhaps as an ally in this ongoing endeavor. Moreover, Yael hints at her cognizance of the importance of her qualifications, acknowledging that they grant her a voice within her religious community. This recognition may be attributed to the prevailing sentiment in Modern Orthodox identity in Israel, where the acknowledgment of the empowering role of secular education is prominent.

About a third of the participants presented similarities between them and the Orthodox-Arab women, such as: "getting married at a young age," "dressing modestly," "giving birth to many children," and more. These participants seem to sympathize with the situation of Orthodox-Arab women and do not criticize them for being religiously conservative. At the same time, in some papers women blamed the Arab women for their inferior social status in society. These participants rejected any resemblance to Arab-Orthodox women and even criticized them and their low status at home and in family life:

We are not like them at all, and there is nothing to compare the status of women in Jewish society to the status of women in Arab society. They do not make any important decisions at home, and even who to marry – they cannot choose. In the Jewish orthodox sector, women also marry at a young age, but choose the husband they love, and then, married life is conducted equally. My husband does not tell me what to wear or where to go. I decide for myself (Hagit).

By comparison the status of women in both societies, Jewish and Arab, Hagit presented a belief in gender equality in marriage and family life, but only for Jewish Orthodox women. That is, according to some participants the Arab women hold traditional views, for example: "I understand them [the Arab women], they do not want to change their situation too much […] And even if there is inequality with men, one should stick to existing customs and not try to change 'world orders" (Rachel).

The pattern of highlighting an approach for equality in gender roles which is applied only to Jewish women while emphasizing the contrast with the Arab women continues by some women (25 %) who related to actual tensions, and expressed frustrations regarding the difficulty of living as a Modern Orthodox woman. Although the papers conveyed respect for Orthodox norms such as motherhood, modesty, and deference to male authority, a central conflict emerged—the tension between adhering to the prescribed Orthodox path for women and the desire to forge their own destinies. Rejecting a likeness to Arab women, Michal outlined a strategic approach as a means of managing this internal strife. Modern Orthodox women establish their distinctiveness through comparisons with two groups. Firstly, they contrast themselves with Arab women, appreciating their relatively greater autonomy. Secondly, they differentiate from those within their community without tertiary education, viewing themselves as 'free' in comparison.

At the same time, Michal describes negotiations she conducts with her family members (mainly with her husband) so that they cooperate with her and help and do not prevent her fulfilling her educational and professional ambitions:

They [the Arab women] seem to me to have no ambitions; most of the Arab female students here did not choose what to study at all, their father or their husband decided for them. For them, the ambition is to sit at home and raise children or work part-time. The main thing is to make money for the family … when I got pregnant immediately after the wedding, I told my husband that I would shorten the maternity leave so as not to miss a school year in the college. True, it was not easy to convince my husband, but I explained to him that he has a career and I have a career, and we will have to find a solution. Now, I can combine studies half a week and a half a week to take care of (my) baby and that way we are both happy. We did not send the baby to the nursery because his mother is helping us, so we saved money […] I'm not against the *Halachic* norms, but it’s a matter of survival […] when you are determined and flexible there is no case that cannot be resolved […] I cannot be that kind of woman, the wife who only sits at home (Michal).

Michal provides insight into the negotiation of roles and responsibilities within a marriage, the evolving dynamics of gender roles in Modern Orthodox family. She emphasizes the importance of negotiation within her marriage in order to achieve her desire to continue her education and career. The statement reflects the modern approach of balancing traditional gender roles with personal and professional aspirations. Michal and other women express a desire to both be a mother and pursue their careers. This illustrates the changing dynamics of gender roles in society. Near the end of her statement, Michal mentions not being against "Halachic norms" (Jewish religious laws) but emphasizes the need for adaptation and survival within her cultural and religious context. Importantly, she emphasizes that this necessity for adaptation is not solely a matter of religious and cultural preservation but also carries economic implications. This shows the complexity of adhering to traditional values while also pursuing personal and professional ambitions. The women's determination and adaptability are evident as they seek solutions to balance their roles as wifies, mothers, and students. This highlights the role of flexibility and determination in these women's identity development.

## 5 Discussion

This study investigated how engagement in a multicultural setting influences the identity development among young Modern Orthodox women who were enrolled in a dialogue course. In order to gain deeper understanding of the participants' identity-related challenges, experiences, and the strategies they utilized for self-exploration within the multicultural context of the study, a content analysis was conducted. This analysis involved a thorough examination of the written papers produced by the participants. The findings of this study indicate that women tried to understand and make sense of how cultural and ethnic differences influenced their own perceptions of themselves and their identity. They were engaging in a process of self-exploration, particularly when they encountered Arab students. Individuals in traditional societies, often express unwillingness to consider changes to their worldview because they have consolidated their identity development without undergoing substantial reflection [45]. However, this study challenges that notion by demonstrating that active participation in dialogue, as well as openly expressing one's thoughts and feelings, can actually have a positive effect on conservative young women. These findings provide additional evidence supporting the idea that identity development is context-dependent. The context of participation in a dialogue course, particularly within a Modern Orthodox group, offers insights into how these young religious women manage their perceptions and identities while respecting their religious and cultural norms.

Researchers argue that intergroup dialogues are an ideal context to study the mutual impact of multiple identities [34]. Therefore, and in accordance with previous research [42], participants concentrated on their group identity, but mainly from a perspective of social comparison to the members of the Arab group, emphasizing the differences between the groups. This exploration strategy can be understood as a means of strengthening women's self-affirmation [46]. This finding is also consistent with Tajfel and Turner's theory (1986) about the centrality of group identity in conflict situations, hence, in light of the Jewish-Arab conflict, participants' ethnic and cultural differences received much attention during the dialogue and after the course [31]. Furthermore, it is possible that the dialogue group provided a safe environment to voice insights that may be taboo outside this group [34]. Thus, participants have focused on group membership and especially, expressed their politicized identity. In particular, they viewed the Arab women in the college as belonging to a different group in the context of the Israeli-Arab conflict [47], which prevented them from recognizing common ground or the potential for collective feminist action in the college.

The study reveals that young Modern Orthodox women face a challenge in balancing their religious beliefs with the modern world, particularly in preserving their traditions while desiring change. According to the findings, these women chose to define themselves as unique as a strategy to cope with this complex reality. In fact, even though the participants expressed strong confidence in the way they lived and the choices they made, they were careful not to challenge boundaries of Jewish law, leadership and ideology (social norms). As a result, an ambivalent identity process was revealed; both a strong sense of self-confidence to be different, and a strong identification with the group and its religious demands. These findings highlight an identity development process among Modern Orthodox women, which moves away from rigid dichotomies towards an understanding of how these women negotiate their complex dimensions of identity [6]. Most of the participants have successfully managed complex identity dynamics, particularly in the context of pursuing higher education and striving for career development. These dynamics involved reconciling various responsibilities and challenges, particularly related to aspects such as career choices, work-family balance [48,49], and their roles within marriage and family dynamics. Despite these challenges, the women are expressing a desire for self-fulfillment, encompassing personal growth. This aligns with earlier findings highlighting that, among the key factors impacting the life satisfaction of Israeli Modern Orthodox women, self-mastery, and positive religious coping strategies play a crucial role [50]. Additionally, they seek equal opportunities, indicating a commitment to gender equality and a desire to overcome traditional gender roles and restrictions [8,51].

Even though terminology may not have been explicitly feminist, the participants were still actively considering and discussing gender-related issues. This indicates a nuanced understanding of gender dynamics and a willingness to explore related topics. Moreover, the findings underscore a dialectical process marked by two contrasting aspects: the adoption of feminist perspectives and adherence to traditional views. Some women seamlessly integrate feminist ideas into their religious beliefs, aligning with earlier research that highlighted the compatibility of feminist perspectives with Modern Orthodox Jewish identity [13,14]. Conversely, other women in the study grapple with dilemmas between religion and feminism, particularly regarding traditional perspectives on the division of family roles and conflicts between home and work. This dialectical process is complemented by a prior finding in a recent study, unveiling the emergence of a distinctive strand of Orthodox feminism within the Israeli Jewish context.

### 5.1 Limitations and future research

The findings of this study may be limited, because they are based on an analysis of a sample of Jewish women only in a specific multicultural academic setting, a dialogue course, potentially affecting the generalizability of the study's findings to a broader population. In particular, the findings may be influenced by the self-selecting nature of the group, which consists of women choosing to attend a non-religious college. Recognizing this limitation, it is suggested that future research endeavors consider incorporating more diverse samples. This could encompass women from religious colleges and those not pursuing higher education, offering a more comprehensive understanding of how identity development operates across diverse religious and non-religious groups.

Another limitation of this study is that the timing of when the reflections were made and written is unknown since the papers were submitted at the end of the course, without information regarding any potential long-term effects. Additionally, the reflective feedback from female students was collected amid notable events in Israeli society, including the ongoing COVID-19 pandemic. Recognizing this temporal gap allows us to consider how these societal changes may have influenced the perspectives and experiences of the individuals studied, enhancing the contextual depth of this research. Hence, further exploration and evaluation of this process is recommended during a longer stage in participants' lives (e.g., a longitudinal study). Further studies should also include different methodical examinations of the concept of identity through interviews and designated tools, as well as other dialogues.

### 5.2 Conclusion

The current research makes a significant contribution to the study of identity development among Modern Orthodox women by presenting strategies of self-exploration as adaptive to different social contexts. Findings revealed how relating to different cultural identities in college encounters provide participants with an opportunity to examine attitudes and self-identifications in relation to their group membership, gender and religion. The multicultural context was conductive to the identity development and thus, highlights the practical implications of the research findings in the context of education and multiculturalism, emphasizing the potential benefits for individuals from diverse backgrounds. Understanding the positive impact of multicultural environments on identity development can inform educational practices and policies aimed at fostering inclusivity and promoting healthy identity exploration among students from various cultural backgrounds [52]. In addition, the research captures a broader range of perspectives on gender issues, going beyond traditional terminology and labels. Thus, the findings have the potential to inform interventions and support systems that can assist these women in managing the complexities of their identities within the confines of their conservative community.

In a broader context, this study contributes significantly to the understanding of identity development within Modern Orthodoxy. It emphasizes that the negotiation of identity goes beyond a simple balancing act between religious and secular values. Instead, it involves a nuanced interplay within the intricate spaces where these negotiations unfold. The findings shed light on the complex tapestry of identity formation for Modern Orthodox individuals, intricately weaving together personal beliefs, social affiliations, and the specific environments in which they grapple with the intricacies of their evolving worldviews.

## Declarations

### Informed participants consent

The author confirms that written informed consent has been obtained from all the participants and, they have given approval for their information to be used in this study.

### Data availability statement

The author does not have permission to share data.

### Declaration of interests statement

The author declares no conflict of interest.

## Credit authors statement

**Lipaz Shamoa-Nir:** Study conception and design, Data collection, Analysis and interpretation of results, and Manuscript preparation.

## Declaration of competing interest

The authors declare that they have no known competing financial interests or personal relationships that could have appeared to influence the work reported in this paper.
